# Supportive treatment using a compression garment vest of painful sternal instability following deep surgical wound infection: a case report

**DOI:** 10.1186/1752-1947-4-266

**Published:** 2010-08-11

**Authors:** Andreas Klement, Manfred Herrmann

**Affiliations:** 1 Institute of General Practice, Martin-Luther-University Halle-Wittenberg, Magdeburger Str. 18, D-06112 Halle, Germany; 2 Department of Cardiac and Thoracic Surgery, University Hospital Halle, Ernst Grube Str. 40, D-06120 Halle, Germany

## Abstract

**Introduction:**

Sternal dehiscence and instability poses a significant cause of persistent pain and limited quality of life following hospital discharge for 0.2% to 5% of patients who have undergone median sternotomy for open heart surgery. We report a successful, conservative, supportive long-term therapy of painful sternal non-union using a customized compression garment vest.

**Case presentation:**

We report a case of painful sternal instability following open heart surgery in a 74-year-old Caucasian man. The complicating factors of obesity (body mass index of 40), renal failure, insulin-dependent diabetes mellitus and absolute arrhythmia with atrial fibrillation were present.

**Conclusion:**

A number of studies have demonstrated the efficacy of surgical interventions for secondary sternal stabilization, but individual patients may reject this option or may be, for other reasons, no longer operable. The task of primary care physicians and other health care providers is to offer this group of patients an alternative option for pragmatic, inexpensive and effective supportive therapy, of which compression garments are an example.

## Introduction

Deep surgical wound infections (DSWIs) after coronary artery bypass grafting (CABG) are known to be rare but serious complications. Sternal dehiscence occurs in 0.2% to 5% of patients who have undergone median sternotomy, and poses a significant cause of persistent pain and limited quality of life following hospital discharge [1]. Although diverse studies have demonstrated the efficacy of surgical interventions for secondary sternal stabilization, individual patients reject this option or are for other reasons no longer operable [2]. The task of primary care clinicians is to offer this group of patients an alternative option for pragmatic and cost-effective conservative therapy.

## Case presentation

**Figure 1:**
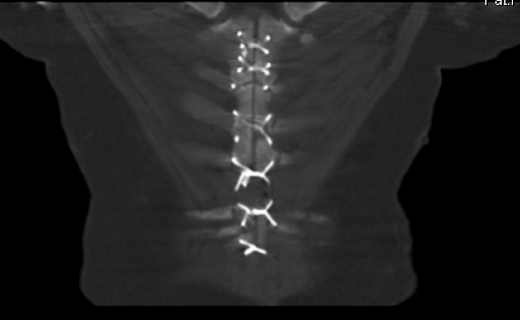
**A computed tomography (CT) scan prior to supportive therapy showing sternal non-union (two months after operative refixation)**.

We report a case of painful sternal instability following combined open aortic valve replacement and CABG in a 74-year-old Caucasian man. Alongside the surgically treated aortic valve stenosis (grade III) and two-vessel coronary disease, the complicating factors of insulin-dependent diabetes mellitus, obesity (body mass index (BMI) of 40), chronic renal insufficiency, and absolute arrhythmia with atrial fibrillation were present. Due to DSWI with confirmation of massive-scale *Staphylococcus epidermidis *infiltration, wound revision, necrectomy, and vacuum-assisted closure (VAC) were necessary. Ultimately after three weeks of VAC, secondary closure of the thorax could be performed. Considerable wound pain associated with breathing, palpable sternal instability, and local indications of inflammation persisted in our patient for a further three months. A computed tomography (CT) scan of the thorax conducted in response showed sternal non-union up to six mm wide, an old, organizing hematoma closely surrounding the sternum in a cloak-like manner with a width up to 25 mm, and intact wire stitches (Figure 1). He rejected a repeat surgical wound revision.

**Figure 2:**
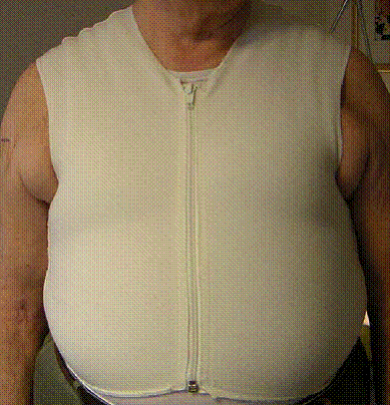
**Customized compression garment vest on our 74-year-old patient with a body mass index (BMI) of 40**. He experienced painful sternal non-union following median sternotomy.

**Figure 3:**
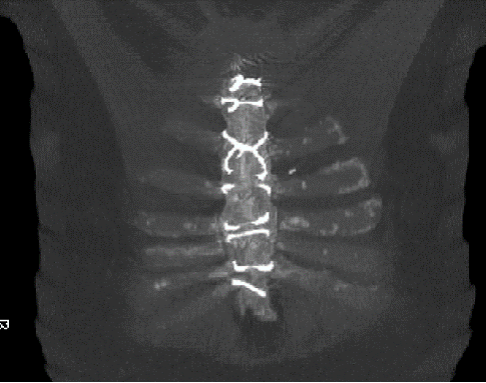
**Coronal computed tomography (CT) scan demonstrating the complete osseous wound healing following three months of wearing a compression garment vest (7 months after operative refixation)**.

We decided in agreement with our patient at his general practitioner's practice to attempt a conservative supportive therapy with an external thoracic support in the form of a customized elastic vest of viscose polyester flat knitwear, with a compression pressure of approximately 20 to 30 mmHg (2.6 to 4 kPa) (Figure 2). Such 'compression garments' have been used for more than 10 years for compression treatment of hypertrophic scarring [3]. Subsequently, the subjective pain intensity decreased within four weeks from a score of eight to nine down to two to three on a visual analog scale of 10 points (100 mm); the need for opioid analgesics was reduced from transdermal fentanyl 100 μg/hour every 72 hours to 12.5 μg/hour every 72 hours. Due to its multi-directional elastic characteristics (approximately 5N/15% fabric stretching), the garment proved itself to be well tolerated even when worn full time under typical everyday conditions. The sternum showed palpatory stability following conservative treatment for three months in total; external scarring was unremarkable. A final CT scan documented completed osseous wound healing and irritation-free connective tissue (Figure 3).

## Conclusion

Devices for external compression of the thorax to prevent or treat sternal instabilities have been systematically investigated in only a few studies, but have shown promising results [4]. Relatively rigid 'corset-like' constructions can, as we have occasionally observed, easily slip out of place, particularly on patients who are obese, and cause skin irritations at the edges of the material due to bulging skin. An alternative is offered by compression garments: they are available worldwide from different manufacturers and in a variety of materials, are relatively inexpensive, and suitable for practical daily use due to their elasticity. In a pilot study involving 15 patients, they were found to be not significantly inferior to rigid 'adjustable fastening braces' in their adaptive effects on sternal wound edges. Long-term comparisons of safety and efficacy in larger study populations have not yet been conducted [5]. To the best of our knowledge, there has been no report to date on conservative supportive long-term therapy of painful sternal non-union using compression garments.

## Consent

Written informed consent was obtained from the patient for publication of this case report and any accompanying images. A copy of the written consent is available for review by the Editor-in-Chief of this journal.

## Competing interests

The authors declare that they have no competing interests.

## Authors' contributions

MH analyzed and interpreted patient data regarding the cardiac and thoracic condition of our patient and the reasons for persisting pain. AK conducted the continuous primary health care, tailoring of the pragmatic supportive therapy and follow-up of our patient, and was a major contributor in writing the manuscript. All authors read and approved the final manuscript.
